# Regulation of PD-L1 expression in the tumor microenvironment

**DOI:** 10.1186/s13045-020-01027-5

**Published:** 2021-01-07

**Authors:** Ming Yi, Mengke Niu, Linping Xu, Suxia Luo, Kongming Wu

**Affiliations:** 1 Department of Oncology, Tongji Hospital of Tongji Medical College, Huazhong University of Science and Technology, Wuhan, 430030 China; 2 Department of Medical Oncology, The Affiliated Cancer Hospital of Zhengzhou University and Henan Cancer Hospital, Zhengzhou, 450008 China

**Keywords:** Cancer immunology, PD-L1, PD-1, Transcriptional regulation, Post-transcriptional modification

## Abstract

Programmed death-ligand 1 (PD-L1) on cancer cells engages with programmed cell death-1 (PD-1) on immune cells, contributing to cancer immune escape. For multiple cancer types, the PD-1/PD-L1 axis is the major speed-limiting step of the anti-cancer immune response. In this context, blocking PD-1/PD-L1 could restore T cells from exhausted status and eradicate cancer cells. However, only a subset of PD-L1 positive patients benefits from α-PD-1/PD-L1 therapies. Actually, PD-L1 expression is regulated by various factors, leading to the diverse significances of PD-L1 positivity. Understanding the mechanisms of PD-L1 regulation is helpful to select patients and enhance the treatment effect. In this review, we focused on PD-L1 regulators at the levels of transcription, post-transcription, post-translation. Besides, we discussed the potential applications of these laboratory findings in the clinic.

## Background

In physiological conditions, the activities of T cells are intricately regulated. T cell immunity selectively eliminates pathogens and abnormal cells but avoids attacking normal cells, termed immune homeostasis [1]. Programmed cell death-1 (PD-1, which is encoded by *PDCD1*) and programmed death-ligand 1 (PD-L1, which is encoded by *CD274*) are vital proteins in maintaining immune homeostasis [2]. The PD-1/PD-L1 pathway restrains the hyperactivation of immune cells and prevents autoimmune diseases [3]. However, in the tumor microenvironment (TME), the PD-1/PD-L1 axis is hijacked by cancer cells to escape immune surveillance [4]. The overexpressed PD-L1 on cancer cells binds to the PD-1 on tumor-infiltrating lymphocytes (TILs), which counteracts the TCR-signaling cascade by phosphorylating SHP-2 [5, 6]. As a result, T cell activation is impaired. Apart from cancer cells, some other types of cells in the TME, such as macrophages, dendritic cells (DCs), activated T cells, as well as cancer-associated fibroblasts, also express PD-L1 [7]. These components orchestrate an immunosuppressive microenvironment, supporting tumor growth.

Inhibiting the PD-1/PD-L1 signaling is a feasible strategy to normalize the dysregulated TME [8]. Up to now, α-PD-1/PD-L1 treatments have exhibited potent antitumor activities in various cancers, such as melanoma, non-small cell lung cancer (NSCLC), gastric cancer, liver cancer, urothelial cancer, lymphoma, and all MSI-high cancers [2, 9–19]. Commonly, the PD-L1 protein level is the primary standard to select patients who are more likely to respond to α-PD-1/PD-L1 treatments [20, 21]. However, the PD-L1 level is determined by several factors, which results in the different significances of PD-L1 positivity or negativity. The PD-L1 positivity might result from immune response-induced PD-L1 expression or oncogenic constructive PD-L1 upregulation [22]. For the latter, in the absence of pre-existing immune response, patients with PD-L1 positive tumors commonly are resistant to α-PD-1/PD-L1 therapies [20].

On the contrary, patients with PD-L1 negative tumors might respond to α-PD-1/PD-L1 treatment when undergoing combination therapies that promote T cell infiltration [22]. Therefore, an in-depth understanding of PD-L1 regulation is valuable for efficacy prediction and patient selection. In this review, we summarized the latest advances of PD-L1 regulation, including genomic alterations, epigenetic modification, transcriptional regulation, post-transcriptional modification, and post-translational modification. Moreover, we discussed the potential applications of these findings in the clinic.

## Genomic alterations of *CD274*

**Fig. 1:**
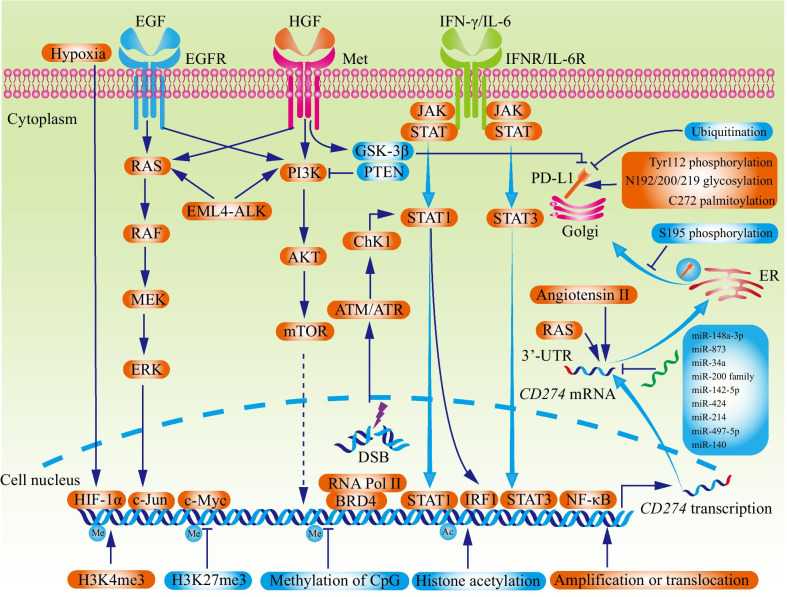
The regulators of PD-L1 expression. PD-L1 abundance is regulated by genomic alterations (amplification or translocation), epigenetic modifications (methylation of histone or CpG island, and histone acetylation), transcriptional regulation (inflammatory stimuli and oncogenic signals), post-transcriptional regulation (miRNA, the status of 3′- UTR, RAS, and Angiotensin II), and post-translational modification (ubiquitination, phosphorylation, glycosylation, palmitoylation). H3K4me3: tri-methylation of histone H3 on lysine 4; H3K27me3: tri-methylation of histone H3 on lysine 27; EGFR: epidermal growth factor receptor; IRF: interferon-responsive factor; IFN: interferon; DSB: double-strand break; GSK3β: glycogen synthase kinase 3β; PI3K: phosphoinositide 3-kinase; NF-κB: Nuclear factor kappa-B; HIF-1α: hypoxia-inducible factor-1α; ALK: Anaplastic lymphoma kinase; ER: endoplasmic reticulum

In some cancers such as classical Hodgkin lymphoma and small-cell lung cancer, the copy number of chromosome 9p24.1 (where *CD274* resides) was increased [23, 24]. The chromosome rearrangement caused *CD274* amplification without influences on the open reading frame (Fig. 1) (Table 1) [24]. Besides, in mediastinal large B-cell lymphoma, the increased transcriptional expression of *CD274* was related to an adjacent ectopic promoter or enhancer by translocation [25]. These findings indicated that genomic alterations contributed substantially to cancer immune escape, which might be a potential biomarker for patient selection.

**Table 1:** The mechanisms of PD-L1 regulation in the tumor microenvironment

Regulatory stage	Regulator	The change of PD-L1	Cancer type	References
Genomic alterations	Gene amplification or translocation	Up	Classical Hodgkin lymphoma; Small-cell lung cancer; Large B-cell lymphoma	[23–25]
Epigenetic regulation	H3K4me3	Up	Pancreatic cancer	[27]
	H3K27me3	Down	Hepatocellular carcinoma	[29]
	Methylation of some CpG loci in *CD274* promoter	Down	Melanoma; Head and neck squamous cell carcinomas; Colorectal cancer	[33–35]
	Histone acetylation	Up	Drug-resistant cancer cells;	[39]
Transcriptional regulation	IFN-α, IFN-β, IFN-γ	Up	Melanoma; Hepatocellular carcinoma; Gastric carcinoma	[44, 46, 48]
	IL-6	Up	Prostate cancer; Hepatocellular carcinoma; Glioblastoma; Non-small cell lung cancer	[50–53]
	TNF-α	Up	Prostate cancer; Colon cancer; Renal cell carcinoma	[54, 55]
	IL-10	Up	Oral squamous cell carcinoma	[56]
	IL-27	Up	Epithelial ovarian cancer; Prostate cancer; Non-small cell lung cancer	[57]
	TGF-β	Up	Lung cancer	[60, 61]
	EGFR	Up	Lung cancer	[62]
	MAPK	Up	Lung cancer; Melanoma; Pancreatic cancer; Triple-negative breast cancer	[67–70]
	PTEN	Down	Triple-negative breast cancer	[74]
	PI3K	Up	Gastric cancer; Her2-overexpressing cell lines; Colorectal cancer; Head and neck squamous cell carcinomas; Non-small cell lung cancer	[75–79]
	JAK-STAT	Up	Natural killer/T-cell lymphoma; Non-small cell lung cancer; Triple-negative breast cancer;	[80–83]
	NF-κB	Up	Natural killer/T-cell lymphoma; Gastric carcinoma; Non-small cell lung cancer; Triple-negative breast cancer	[84, 87–90]
	HIF-1	Up	Prostate cancer; Breast cancer; Nasopharyngeal carcinoma	[92, 93]
	Myc	Up	Leukemia and lymphomas; Melanoma; Non-small cell lung cancer; Hepatocellular carcinoma; Renal cell carcinoma; Colorectal carcinoma; Esophageal squamous cell carcinoma; Pancreatic cancer	[96–99]
	ALK	Up	Non-small cell lung cancer; Anaplastic large cell lymphoma	[103, 104]
	Met	Up	Non-small cell lung cancer	[107–110]
	BRD4	Up	Ovarian cancer	[112]
	DSB	Up	Osteosarcoma; Non-small cell lung cancer; Prostate cancer	[114]
Post-transcriptional regulation	miR-148a-3p	Down	Colorectal cancer	[116]
	miR-873	Down	Breast cancer	[117]
	miR-34a	Down	B-cell lymphoma; Acute myeloid leukemia	[118, 126]
	miR-200 family	Down	Non-small cell lung cancer; Hepatocellular carcinoma	[119, 120]
	miR-142-5p	Down	Pancreatic cancer	[121]
	miR-424	Down	Ovarian cancer	[122]
	miR-214	Down	Diffuse large B-cell lymphoma	[123]
	miR-497-5p	Down	Clear cell renal cell carcinoma	[124]
	miR-140	Down	Non-small cell lung cancer	[125]
	miR-23a-3p/PTEN axis	Up	Liver Cancer	[127]
	miR-200a/PTEN axis	Up	Osteosarcoma	[128]
	miR-27a-3p/ MAGI2/PTEN axis	Up	Breast cancer	[129]
	miR-145/c-Myc axis	Down	Ovarian cancer	[130]
	miR-18a/ PTEN, WNK2, SOX6 axis	Up	Cervical cancer	[131]
	miR-BART5/PIAS3/pSTAT3	Up	Gastric cancer	[133]
	RAS-tristetraprolin	Up	RAS mutant cancer	[135]
	Angiotensin II/human antigen R	Up	Non-small cell lung cancer	[136]
Post-translational modification	Ubiquitination	Down	Multiple cancers	[138, 141, 142]
	Y112 phosphorylation	Up	Hepatocellular carcinoma	[143]
	S195 phosphorylation	Down	Breast cancer	[144]
	T180 and S184 phosphorylation	Down	Breast cancer	[145]
	Glycosylation	Up	Breast cancer; Glioma	[145, 149, 150]
	Palmitoylation	Up	Breast cancer; Colon cancer	[151, 152]

*H3K4me3* tri-methylation of histone H3 on lysine 4, *H3K27me3* tri-methylation of histone H3 on lysine 27, *EGFR* epidermal growth factor receptor, *MAPK* mitogen-activated protein kinase, *PTEN* phosphatase and tensin homolog, *PI3K* phosphoinositide 3-kinase, *NF-κB* nuclear factor kappa-B, *HIF-1α* hypoxia-inducible factor-1α, *ALK* anaplastic lymphoma kinase, *DSB* double-strand break

## Epigenetic regulations

Epigenetic regulations such as methylation and histone acetylation determine the PD-L1 expression as well (Fig. 1). Tri-methylation of histone H3 on lysine 4 (H3K4me3) is generally believed as a histone modification promoting gene transcriptions [26]. In pancreatic cancer, MLL1 protein could bind to the *CD274* promoter to catalyze H3K4me3, leading to the increased expression of PD-L1 [27]. In agreement, the MLL1 inhibitor had a synergistic effect with α-PD-1/PD-L1 therapy [27]. On the contrary, tri-methylation of histone H3 on lysine 27 (H3K27me3) relates to transcription suppression [28]. In hepatocellular carcinoma, enhancer of zeste homolog 2 negatively regulated PD-L1 expression by promoting H3K27me3 [29].

Besides the methylation of histone, the methylation of DNA at CpG islands regulated PD-L1 expression [30]. Inhibiting methylation of DNA by DNA methyltransferase inhibitors (DNMTis) increased PD-L1 level in cancer cells [31, 32]. The authors assumed that DNMTis elevated the expression of hypermethylated endogenous retroviruses in cancer cells, which might activate the innate immune response and lead to IFN-γ-stimulated PD-L1 expression [30]. Moreover, the methylation of some specific CpG loci in the *CD274* promoter determined the level of *CD274* mRNA [33–35]. In NSCLC, TGF-β1 impaired the activity of DNMTs, demethylated the *CD274* promoter, and increased PD-L1 expression [36]. Notably, in patients with recurrent gastric cardia adenocarcinoma, PD-L1 expression was reduced after α-PD-1/PD-L1 treatment [37]. Further investigations indicated that the *CD274* promoter was more hypermethylated in the relapsed tumors than in the primary tumors without α-PD-1/PD-L1 treatment [37]. In murine tumor models, the combination therapy of hypomethylating agent azacytidine and α-PD-1 showed a more significant antitumor effect than α-PD-1 monotherapy [37].

Histone acetylation is an epigenetic modification enhancing gene transcription [38]. In some drug-resistant cancer cells, hyperactivated JNK/c-Jun signaling suppressed the histone deacetylase 3 (HADC3) expression, thereby elevating the histone H3 acetylation of the *CD274* promoter [39]. The HADC inhibitor had a synergistic effect with α-PD-1 in the B16F10 tumor model [40]. Furthermore, HADC inhibitor-mediated PD-L1 upregulation was observed in other types of cancers [41, 42]. These findings provide a rationale to combine α-PD-1/PD-L1 treatments with HDAC inhibitors.

## Transcriptional regulation

### Inflammatory Signaling

#### Interferon (IFN) and IL-6

As a negative feedback for inflammation, PD-L1 could be upregulated by multiple inflammatory signaling pathways to restrain T cells' hyperactivity (Fig. 1). Generally believed, IFN-γ is the prominent stimulator contributing to the inducible PD-L1 expression [43].

During cancer progression, the IFN-γ-derived PD-L1 promotes cancer immune escape [3]. In the TME, activated T cells and NK cells generate most IFN-γ. Then, IFN-γ binds to type II interferon receptor, activating the JAK-STAT signaling (mainly through STAT1) [44, 45]. Subsequently, the expression of several transcriptional factors is upregulated, especially interferon-responsive factors (IRFs). IRF-1 is the vital downstream component of STAT1 upon IFN-γ treatment [46, 47]. In hepatocellular carcinoma, it was identified that two elements (IRE1/2) in the 5′-flanking region of the *CD274* promoter were the binding sites of IRF-1, which participated in regulating PD-L1 transcription [48]. Notably, the intactness of JAK-STAT-IRF1 pathway is also related to the response to α-PD-1/PD-L1 therapy. The effect of α-PD-1/PD-L1 treatment is limited in tumors with mutations in *JAK1* and *JAK2* [49]. It was speculated that these tumors might not rely on the PD-1/PD-L1 pathway to escape immune surveillance [49].

Besides IFN-γ, other inflammatory stimuli such as IFN-α, IFN-β, and IL-6 could induce PD-L1 expression as well. However, IFN-α and IFN-β had a more significant effect on PD-L2 regulation than PD-L1 regulation [44]. In prostate cancer, the IL-6-JAK-STAT3 pathway promoted PD-L1 expression and led to the resistance to immune killing [50]. Moreover, in hepatocellular carcinoma, increased IL-6 activated the STAT3/c-MYC/miR-25-3p pathway, which resulted in the decreased protein tyrosine phosphatase receptor type O (PTPRO) [51]. The downregulated PTPRO-enhanced PD-L1 expression by deregulating the activation of JAK2-STAT1/3 [51]. Furthermore, the glioblastoma-derived IL-6 could induce the local and systemic myeloid PD-L1 expression by STAT3 phosphorylation [52]. Besides, in lung cancer, it was detected that IL-6-derived PD-L1 expression was related to multiple pathways, especially the MEK-ERK signaling [53].

#### Other inflammatory signals

Tumor necrosis factor-α (TNF-α) increased *CD274* mRNA by activating nuclear factor kappa-B (NF-κB) pathway [54]. In renal cell carcinoma, TNF-α cooperated with IL-4 to enhance *CD274* transcription by activating NF-κB, IκB, and STAT6 [55]. Moreover, in oral squamous cell carcinoma, the IL-10 level in the TME was positively correlated to the abundance of PD-L1 on tumor-associated macrophages [56]. Blocking IL-10 suppressed PD-L1 expression [56]. Furthermore, in several human cancer cells, IL-27 increased *CD274* transcription by promoting the tyrosine phosphorylation of STAT1 and STAT3 [57].

The effect of TGF-β on PD-L1 regulation is still unclear. Although some previous studies indicated that TGF-β downregulated PD-L1 expression in renal tubular epithelial cells and monocytes [58, 59], TGF-β mainly had a positive impact on the PD-L1 expression in the TME. In NSCLC cells, exogenous TGF-β increased the *CD274* transcription probably by Smad-binding elements [60]. The expression of phosphorylated-Smad2 was significantly increased in PD-L1 positive NSCLC patients [60]. Apart from cancer cells, TGF-β could increase PD-L1 expression on DCs in the TME [61].

### Oncogenic Signaling

Besides inflammatory stimuli, growing evidence suggests that hyperactive oncogenic pathways play a vital role in PD-L1 expression (Fig. 1). Therefore, α-PD-1/PD-L1 therapies might have a synergistic effect with oncogenic signal-targeting treatments.

#### Epidermal Growth Factor Receptor (EGFR)

In lung epithelial cells, the mutated EGFR pathway (EGFR T790M) increased PD-L1 expression [62]. For lung cancer cells, PD-L1 expression was impaired after EGFR tyrosine kinase inhibitor (TKI) treatment [62]. In murine EGFR-driving lung cancer models, α-PD-1 effectively reversed T cell exhaustion and retarded tumor growth [62]. The results indicated that the mutant EGFR pathway facilitated tumor to escape from immune surveillance [62]. However, a clinical study showed that EGFR-mutant NSCLC patients tended to resist α-PD-1 therapy [63]. The authors found that although some EGFR-mutant NSCLCs were PD-L1 positive, the concurrent PD-L1 upregulation and abundant TILs were rare [63]. The lack of a pre-existing inflammatory TME might limit the effect of α-PD-1/PD-L1 treatment [63]. The low response rate in EGFR-mutant patients was reported by other investigators [64, 65].

#### Mitogen-activated protein kinase (MAPK)

MAPK is a well-studied oncogenic pathway, which counts for nearly 40% of human cancer cases [66]. According to TCGA database, the *CD274* mRNA level was significantly positively related to RAS- or MEK-activation scores in NSCLC patients [67]. In lung adenocarcinoma cells, activating EGF-MAPK signaling increased the mRNA and protein levels of PD-L1 [67]. Inhibiting MAPK signaling by MEK inhibitor (Selumetinib) counteracted the EGF- and IFN-γ-stimulated upregulation of *CD274* mRNA and PD-L1 protein [67]. In melanoma cells, the activated NRAS-RAF-MEK1/2-ERK-c-Jun axis enhanced the transcription of *CD274* [68]. Moreover, in pancreatic cancer, myeloid cells induced PD-L1 expression on tumor cells by activating EGFR-MAPK pathway [69]. After MEK inhibitor treatment, the levels of p-ERK and PD-L1 were decreased, and this reduced PD-L1 led to a higher sensitivity to α-PD-1 treatment in murine pancreatic tumors [69].

On the contrary, in murine breast cancer cells, suppressing MAPK signaling by Trametinib (a MEK inhibitor) potentiated the IFN-γ-stimulated upregulation of PD-L1 and major histocompatibility complex (MHC) [70]. Furthermore, in cancer cell lines, including KYSE30, TE-1, MKN7, PC-9, SNU-475, OE19, and BT-549, there was no significant alteration when cancer cells were treated with MAPK inhibitor [71]. Besides, the MAPK inhibitor had no significant impact on IFN-γ-stimulated PD-L1 expression [71]. The role of MAPK pathway in PD-L1 regulation might depend on cell types [72].

#### PTEN/PI3K-AKT pathway

As a well-studied tumor suppressor, PTEN is a vital regulator of the oncogenic signaling pathway PI3K-AKT [73]. PTEN loss and PI3K activation have been identified in multiple types of cancers, including hepatocellular carcinoma, prostate cancer, and breast cancer [73]. Deficient PTEN was detected in nearly half of PD-L1 positive triple-negative breast cancer samples [74]. Knocking down PTEN resulted in a rise of PD-L1 expression [74]. Moreover, activating the PI3K-AKT pathway in gastric cancer cells increased PD-L1 abundance, while PI3K inhibitor (LY294002) reduced PD-L1 level [75]. Besides, in head and neck cancer cells, melanoma cells, colorectal cancer cells, and Her2-amplified cancer cells (SNU216, NCI-N87, and SKBR3), PD-L1 expression was suppressed by PI3K inhibition [68, 76–78]. Moreover, inhibiting mTOR (the downstream of PI3K-AKT) by rapamycin reduced PD-L1 level in NSCLC cells [79].

#### JAK-STAT pathway

Mutations in *JAK1*, *JAK3*, and *STAT3* were prevalent in mature T-cell lymphomas [80]. Some *STAT3* mutations, such as p.E616K, increased the STAT3 phosphorylation and STAT3-mediated transcription [80]. In the meanwhile, silencing STAT3 or STAT3 inhibitor reduced PD-L1 expression [80]. Further chromatin immunoprecipitation qPCR assay indicated the p.E616K mutation might increase the transcription activity of *CD274* promoter by a stronger STAT3 binding [80]. Besides, in breast and lung cancer cells, the PD-L1 expression was hampered by JAK and STAT3 inhibitors [81–83].

#### NF-κB pathway

Activated NF-κB signaling was related to the high level of PD-L1 in several cancers [36, 84–88]. Multiple oncogenic signals could impair immune surveillance by activating the NF-κB-PD-L1 axis. In lung cancer cells, overexpressed MUC1-C increased the occupancy of NF-κB p65 in *CD274* promoter, which enhanced *CD274* transcription [89]. Besides, in breast cancer, reactive oxygen species (ROS) inducers (paclitaxel, glutathione synthesis inhibitor, and buthionine sulphoximine) led to the accumulation of ROS, subsequently activating the downstream NF-κB pathway [90]. In a murine breast cancer model, paclitaxel treatment induced PD-L1 upregulation in tumor-associated macrophages by the NF-κB p65-PD-L1 pathway [90].

#### Hypoxia-inducible factor-1 (HIF-1)

Hypoxia facilitates the drug resistance and distant metastasis of tumor cells [91]. Besides, a hypoxic TME undermines host immunity activities and contributes to immune escape [92]. Hypoxia upregulated PD-L1 expression by HIF-1α [92]. The hypoxia-induced upregulation of *CD274* mRNA was hampered when HIF-1α was silenced [92]. Further investigations suggested the cellular colocalization of PD-L1 and HIF-1α [92]. In the meanwhile, inhibiting HIF-1 signaling could reduce PD-L1 expression in multiple types of cancers [93, 94].

#### Myc

As a transcription factor regulating cell differentiation, proliferation, and apoptosis, Myc is overexpressed in various cancers [95]. Knocking down or inhibiting Myc in cancer cells reduced *CD274* mRNA and PD-L1 protein [96–99]. The results of the ChIP-seq assay showed that Myc could bind to the *CD274* promoter [96]. However, in some particular types of cancer, Myc negatively regulated PD-L1 expression. In hepatocellular carcinoma cells, inhibiting Myc increased the IFN-γ-stimulated PD-L1 expression [100]. Besides, in the murine MycCaP tumor model, Myc inhibitor treatment promoted T cell infiltration, enhanced antitumor immune response, but simultaneously upregulated PD-L1 expression [101]. This PD-L1 upregulation was induced by immune response [101].

#### Anaplastic lymphoma kinase (ALK)

Chromosomal rearrangements in the *ALK* gene are an oncogenic driver for NSCLC [102]. In various NSCLC cell lines, the *CD274* mRNA and PD-L1 protein levels were higher in cells with echinoderm microtubule-associated protein-like 4 (*EML4*)-*ALK* fusion [103]. Ectopic expressing EML4-ALK protein or blocking ALK phosphorylation positively or negatively regulated PD-L1 expression [103]. Besides, inhibiting PI3K-AKT or MEK-ERK pathways reversed the EML4-ALK-induced PD-L1 expression [103]. Apart from NSCLC, the PD-L1 level was higher in ALK-positive systemic anaplastic large cell lymphoma [104].

#### Met

Alterations in the *Met* gene were reported in multiple types of cancers [105, 106]. In primary lung cancer tissues, the level of PD-L1 was positively correlated to the *Met*-amplification [107, 108]. In a microarray assay, inhibiting or knocking down Met substantially reshaped the expression of several immune-related genes, including *CD274* [109]. On the contrary, activating Met by hepatocyte growth factor increased PD-L1 expression [109, 110].

#### BRD4

As a member of the bromodomain and extraterminal (BET) family, BRD4 acts as a super-enhancer of oncogenes [111]. In ovarian cancer cells, BET inhibitor reduced PD-L1 expression in a time- and dose-dependent manner [112]. Further, the ChIP assay showed a significant association of *CD274* promoter and BRD4 [112]. After BET inhibitor treatment, the associations of *CD274* promoter-BRD4 and *CD274* promoter-RNA Pol II were decreased, which contributed to the downregulated *CD274* transcription [112]. Besides, it was validated that BET inhibitor suppressed *CD274* transcription by reducing the BRD4 occupancy at *CD274* promoter, independent of c-Myc [113].

### DNA double-strand break (DSB) repair pathway

After inducing DSB by ionizing radiation, PD-L1 expression was increased in multiple cancer cell lines [114]. In contrast, paclitaxel (a non-DNA damaging agent) treatment had no significant impact on PD-L1 expression [114]. DSB-activated ATM-ATR-ChK1-STAT1/3-IRF1 pathway led to the downstream PD-L1 upregulation [114].

## Post-transcriptional regulation

### microRNA (miRNA)

Cancer-derived miRNA is a vital post-transcriptional regulator for PD-L1 expression in the TME (Fig. 1) [115]. In colorectal cancer cells with mismatch repair deficiency or microsatellite instability-high, miR-148a-3p was decreased while PD-L1 was increased [116]. The results of the co-transfection of miR-148a-3p mimic and wild-type or mutant *CD274* 3′-untranslated region (UTR) luciferase reporter indicated that *CD274* mRNA was the direct target of miR-148a-3p [116]. Furthermore, in breast cancer cells, miR-873 suppressed PD-L1 expression by targeting *CD274* mRNA [117]. Up to now, it was identified that *CD274* mRNA was the direct target of multiple oncogenic miRNAs such as miR-34a, miR-200 family, miR-142-5p, miR-424, miR-214, miR-497-5p, miR-140 [118–126].

Besides, some cancer-derived miRNAs indirectly regulated PD-L1 expression [127–129]. In ovarian carcinoma cells, miR-145 downregulated PD-L1 by targeting *c-Myc* [130]. In cervical cancer cells, increased PD-L1 was related to the upregulation of miR-18a [131]. miR-18a promoted PD-L1 expression by targeting *PTEN* (inhibitor of PI3K-AKT), *WNK2* (inhibitor of MAPK), and *SOX6* (inhibitor of Wnt/β-catenin) [131]. Similarly, hepatocellular carcinoma cell-derived miR-23a-3p enhanced PD-L1 expression in macrophages via targeting *PTEN* [127]. In NSCLC cells, miR-3127-5p promoted PD-L1 expression by activating STAT3 [132]. Moreover, in gastric cancer, miR-BART5-5p increased PD-L1 by targeting *PIAS3* (inhibitor of STAT3) [133].

### The stability of *CD274* mRNA

The variations in the 3′- UTR affected the stability of *CD274* mRNA [134]. Disturbing the 3′-UTR of *CD274* mRNA by Crisper-Cas9 could stabilize *CD274* mRNA [134]. Besides, oncogenic RAS activation inhibited tristetraprolin (AU-rich element-binding protein) by kinase MK2, stabilizing *CD274* mRNA [135]. As a result, RAS activation increased PD-L1 expressed on cancer cells [135]. Moreover, in NSCLC, Angiotensin II increased the stability of *CD274* mRNA and induced PD-L1 expression by human antigen R (also known as HuR, an AU-rich element-binding protein) [136].

## Post-translational modification

Post-translational modifications, including ubiquitination, phosphorylation, glycosylation, palmitoylation, and SUMOylation, play a vital role in regulating protein stability, activation, localization, as well as interaction [137]. Aberrant post-translational modification patterns participated in PD-L1 upregulation in the TME (Fig. 1) [138].

### Ubiquitination

Ubiquitination is related to proteasome-mediated protein degradation [139]. In a broad range of cancer cells, CMTM6 maintained PD-L1 expression by reducing PD-L1 ubiquitination and increasing PD-L1 half-life [138, 140]. Moreover, cyclin D/cyclin-dependent kinase 4 (CDK4) promoted PD-L1 ubiquitination by SPOP/Cullin 3-SPOP E3 ligase [141]. CDK4/6 inhibitor treatment increased PD-L1 abundance, which provided a potential for the combining therapy of CDK4/6 inhibitors and α-PD-1/PD-L1 agents [141]. Besides, the TNF-α-NF-κB pathway inhibited PD-L1 ubiquitination via upregulating COP9 signalosome 5 (CSN5) [142]. Inhibiting CSN5 impaired PD-L1 expression and sensitized tumor cells to the following immunotherapy [142].

### Phosphorylation

IL-6-activated JAK1 promoted the phosphorylation of PD-L1 protein (Tyr112) [143]. Subsequently, Tyr112-phosphorylated PD-L1 recruited STT3A (N-glycosyltransferase) to catalyze the PD-L1 glycosylation [143]. Activating the IL-6-JAK1 signaling elevated PD-L1 stability by this phosphorylation modification [143]. Blocking the IL-6-JAK1 axis had a synergistic effect with α-Tim-3 treatment in murine tumor models [143]. Besides, metformin-activated AMP-activated protein kinase promoted the phosphorylation of PD-L1 (S195) [144]. The S195 phosphorylation led to the aberrant PD-L1 glycosylation, which undermined the PD-L1 translocation from endoplasmic reticulum to Golgi [144]. Apart from hampering the translocation of PD-L1 to cell membrane, the S195 phosphorylation enhanced endoplasmic reticulum-associated PD-L1 degradation [144]. The combination therapy of metformin and α-cytotoxic T Lymphocyte antigen 4 (CTLA-4) exhibited a robust antitumor activity [144]. Moreover, glycogen synthase kinase 3β (GSK3β) decreased the level of PD-L1 by promoting phosphorylation-dependent proteasome degradation [145, 146].

### Glycosylation

Glycosylation modification is related to protein stability [147, 148]. The N192/200/219 glycosylation stabilized PD-L1 and suppressed the formation of GSK3β-β-TrCP-PD-L1 complex [145]. EGF increased PD-L1 expression by promoting glycosylation-induced GSK3β inactivation [145]. Additionally, in epithelial-mesenchymal transition, β-catenin transcriptionally induced the expression of N-glycosyltransferase STT3. The STT3 promoted PD-L1 N-glycosylation, stabilizing and upregulating PD-L1 [149]. Moreover, in glioma, FKBP51s (a co-chaperone) regulated PD-L1 expression by promoting glycosylation modification [150]. Overexpressing or silencing FKBP51s increased or decreased the level of glycosylated-PD-L1 [150].

### Palmitoylation

Palmitoylation is a well-studied post-translational lipid modification. Palmitoylation at C272 increased PD-L1 stability by counteracting its ubiquitination [151, 152]. DHHC3 catalyzed C272 palmitoylation of PD-L1 [152]. Silencing DHHC3 enhanced antitumor immune response in vitro and in vivo [152].

## Perspectives and conclusion

A growing body of evidence suggests that it is inaccurate to select patients merely by PD-L1 abundance. Understanding the difference between inflammation-induced PD-L1 and oncogenic signal-mediated constitutive PD-L1 is helpful to patient selection. For instance, for *EGFR* mutant NSCLC patients, α-PD-1 therapy's efficacy was poor despite the high level of PD-L1 [153]. The *EGFR* mutation-driving NSCLCs commonly harbor lower mutation burdens, and the lower immunogenicity leads to the resistance to α-PD-1 treatments [43]. This oncogenic EGFR-mediated PD-L1 expression could not reflect the real status of the TME. Alternatively, a comprehensive framework containing multiple surrogate markers such as tumor mutation burden would be valuable for selecting patients and predicting outcomes.

**Fig. 2:**
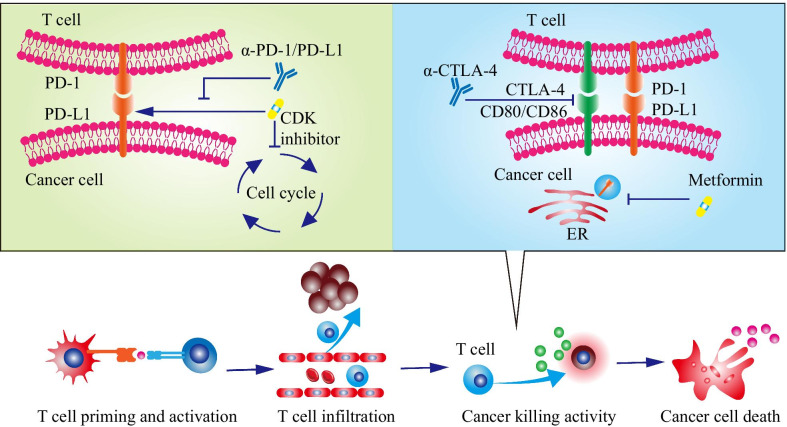
The current immune checkpoint inhibitor therapy could be further enhanced by regulating PD-L1 expression. Agents regulating PD-L1 expression might have a synergistic effect with the current immune checkpoint inhibitors. For example, targeting therapies such as CDK4/6 inhibitor upregulated PD-L1 expression and promoted immune escape. This treatment-induced immune evasion could be overcome by combination therapies containing α-PD-1/PD-L1 (the left panel). Besides, adjuvant treatment regulating PD-L1 expression might elevate the sensitivity to α-PD-1/PD-L1 or other immune checkpoint inhibitors. For instance, metformin downregulated PD-L1 by promoting endoplasmic-reticulum-associated degradation, and the combination therapy of metformin and α-CTLA-4 had a synergistic antitumor activity (the right panel). CDK: cyclin-dependent kinase; CTLA-4: cytotoxic T Lymphocyte antigen 4; ER: endoplasmic reticulum

Besides, agents regulating PD-L1 expression might have a synergistic effect with the current immune checkpoint inhibitors (Fig. 2). Targeting therapies such as CDK4/6 inhibitor upregulated PD-L1 expression and promoted immune escape [141, 146]. This treatment-induced immune evasion could be overcome by combination therapies containing α-PD-1/PD-L1. Besides, adjuvant treatment regulating PD-L1 expression might elevate the sensitivity to α-PD-1/PD-L1 or other immune checkpoint inhibitors [144, 145]. For example, metformin downregulated PD-L1 by promoting endoplasmic-reticulum-associated degradation, and the combination therapy of metformin and α-CTLA-4 exhibited a synergistic antitumor activity [144].

Generally, in the TME, the expression of PD-L1 is regulated by numerous factors, including inflammatory stimuli and oncogenic pathways at the levels of transcription, post-transcription, and post-translation. Exploring potential PD-L1 regulators helps select patients and overcome resistance to α-PD-1/PD-L1 treatments. Besides, the agents regulating PD-L1 expression might be possible adjuvant therapies for the current immune checkpoint inhibitors.

## Data Availability

Not applicable.

## Abbreviations

PD-1: Programmed cell death-1
PD-L1: Programmed death-ligand 1
TME: Tumor microenvironment
NSCLC: Non-small cell lung cancer
H3K4me3: Tri-methylation of histone H3 on lysine 4
H3K27me3: Tri-methylation of histone H3 on lysine 27
EZH2: Enhancer of zeste homolog 2
DNMTi: DNA methyltransferase inhibitors
HADC3: Histone deacetylase 3
IRF: Interferon-responsive factor
IFN: Interferon
PTPRO: Protein tyrosine phosphatase receptor type O
TNF-α: Tumor necrosis factor-α
EGFR: Epidermal growth factor receptor
TKI: Tyrosine kinase inhibitor
MAPK: Mitogen-activated protein kinase
MHC: Major histocompatibility complex
PTEN: Phosphatase and tensin homolog
PI3K: Phosphoinositide 3-kinase
NF-κB: Nuclear factor kappa-B
ROS: Reactive oxygen species
HIF-1α: Hypoxia-inducible factor-1α
ALK: Anaplastic lymphoma kinase
EML4: Echinoderm microtubule-associated protein-like 4
BET: Bromodomain and extraterminal
DSB: Double-strand break
miRNA: MicroRNA
UTR: Untranslated region
CDK: Cyclin-dependent kinase
CTLA-4: Cytotoxic T Lymphocyte antigen 4
GSK3β: Glycogen synthase kinase 3β
